# Janus Magnetic Polymeric Colloids Gradient Thin Films of Amino Dextran Coated Core–Shell Poly (Styrene/Divinylbenzene/Methacrylic Acid) for Ultrasensitive Magnetic Resonance Imaging

**DOI:** 10.1155/ijbm/6630827

**Published:** 2025-10-20

**Authors:** Sundas Khalid, Rafay Naseer, Aqsa Zaheen, Mudassara Saqib, Naveed Ahmed, Abdelhamid Elaissari, Asad Ullah Khan, Kashif Mairaj Deen, Nauman Naseer, Nasir M. Ahmad

**Affiliations:** ^1^Polymer Research Lab, School of Chemical and Material Engineering, National University of Sciences and Technology, Sector H-12, Islamabad 44000, Pakistan; ^2^Weill Cornell Medicine-Qatar, Qatar Foundation, Education City, P.O. Box 24144, Doha, Qatar; ^3^Department of Pharmacology and Therapeutics, Federal Postgraduate Medical Institute (FPGMI), Sheikh Zayed Medical Complex, Lahore, Pakistan; ^4^Department of Pharmacy, Quaid-i-Azam University, Islamabad, Pakistan; ^5^CNRS, ISA, UMR5280, Universite Claude Bernard Lyon 1, 5 rue de la Doua, Villeurbanne 6910, France; ^6^Department of Materials Engineering, The University of British Columbia, Vancouver V6T 1Z4, British Columbia, Canada; ^7^Bahria International Hospital, Takbeer Block, Sector B, Bahria Town, Lahore, Pakistan

**Keywords:** gradient thin films, in vitro diagnosis, iron oxide, lab-on-chip, layer-by-layer, MRI phantoms, superparamagnetic

## Abstract

The present study focuses on developing novel gradient thin films for surface-based magnetic resonance imaging of fluids such as water. Four types of magnetic-polymer colloids were investigated as T2 contrast agents, including Janus magnetic-polystyrene and core–shell magnetic-poly(styrene/divinylbenzene/methacrylic acid) particles. These colloids were coated with amino dextran to enhance their performance. Key factors such as emulsion composition, particle size, and surface properties were systematically examined. Gradient thin films were fabricated on glass slides using a layer-by-layer self-assembled multilayer (LbL-SAMu) technique. The films consisted of positively charged poly(dimethyl diallyl ammonium chloride) and negatively charged magnetic-polymer colloids. The developed colloids and thin films were characterized by their surface wettability, surface morphology, and zeta potential. These films exhibited relatively improved hydrophilicity and T2 contrast. The utilization of such gradient thin films as molecular probes could enhance clinical MRI for in vitro diagnosis. This study indicated that thin-film gradients can offer a facile technique for unique cellular imaging via a lab-on-chip device to enable effective point-of-care molecular diagnostics.

## 1. Introduction

Magnetic resonance imaging (MRI) has become a valuable diagnostic method since its initial clinical use in 1977. An MRI scan provides a detailed image of the organs and tissues of a live subject. It is typically a map of the relative spatial distribution of different molecules, particularly water. A high-contrast image is generated when hydrogen nuclei of water molecules interact with a radio frequency pulse applied perpendicular to a uniform magnetic field [1]. In addition to imaging living subjects, MRI can also be employed as an in vitro diagnostic tool for biological fluids, such as imaging cells, ex vivo histological samples, and individual molecules [2].

The typical resolution of a 1.5 T clinical MRI machine falls within the millimeter-to-sub-millimeter range, which proves to be adequate for achieving a clinical diagnosis [3]. However, if the same machine is to be employed for any one of the in vitro diagnostic applications, enhancement of contrast is needed to improve sensitivity, spatial resolution, and signal clarity [4]. The sensitivity of an MRI scan can be enhanced by different strategies, such as improving machine design [5], developing exogenous contrast agents or molecular probes [6], creating MRI phantoms [7], and augmenting with other nuclear magnetic resonance (NMR) techniques like magnetic resonance microscopy and magnetic resonance force microscopy [7].

Out of all the available strategies, the development of viable contrast agents seems like a feasible strategy to provide a molecular imaging modality to a standard clinical MRI machine [8]. A contrast-enhancing molecule can increase the sensitivity of an image without changing the machine itself. The invention of MRI phantoms for machine calibration has resulted in the design of general protocols for cellular imaging [9]. Some protocols require the incubation of cell suspensions in carved agarose blocks, while others suggest mixing and incubating cell suspensions with varying concentrations of contrast agents [8].

Although easy to perform, cumbersome sample preparation steps limit the use of MRI for rapid, point-of-care diagnostic applications [10]. Most in vitro molecular detection assays using MRI rely on the unimpeded interaction of contrast agents with the target molecule, where uneven dispersion and agglomeration may result in inconsistent signal intensity [11].

Modern research on contrast agents is concentrated on the development of gadolinium-based contrast agents (GBCAs), superparamagnetic iron oxide nanoparticles (Fe3O4/*γ* -Fe2O3) [12], and chemical-exchange-dependent saturation transfer (CEST) [13]. Both T1 (spin-lattice or longitudinal relaxation time) and T2 (spin–spin or transverse relaxation time) contrast agents can be used for image acquisition [14]. However, GBCA produces images with considerably lower signal-to-noise ratios when compared to agents based on iron oxide [15]. Consequently, contrast agents based on iron oxide demonstrate superior sensitivity overall. Typically, the iron oxide-based contrast agents are engineered with a core–shell structure [16]. The size of the magnetic core influences the magnetic response, and the polymeric shell imparts stability [12, 17]. The iron oxide core–shell nanoparticles exhibited negative contrast in T2-weighted MRI, making them suitable candidates for imaging of cells and diagnostic uses within a laboratory [18]. Numerous assays have been developed for the detection of targeted molecules like oligonucleotides, polypeptides, active proteins, saccharides, malignant cells, and various microbes [19].

The electrostatic layer-by-layer self-assembled multilayer (LbL-SAMu) method is an efficient approach for creating multilayered thin films of oppositely charged species on a diverse range of substrates [20]. This technique is advantageous for its simple equipment, short processing time, environmentally friendly approach, and uniform coating on multiple substrates like glass, silicon wafers, and mica. Gorin et al. [21] developed LbL-SAMu thin films of polyethyleneimine and iron oxide nanoparticles on silicon wafers and reported controllable optical and magnetic properties in a variable number of bilayers. Paterno et al. [22] developed electrically and magnetically active LbL-SAMu thin films using poly (o-ethoxy aniline) (POEA), sulfonated polystyrene (PSS), and positively charged maghemite nanoparticles on glass substrates. These films have potential applications in electromagnetic interference shielding and chemical sensing. Hassan et al. [23] fabricated thin films of nickel ferrite nanoparticles and chitosan on glass slides using LbL-SAMu, adopting a manual fabrication protocol. Likewise, thin films of magnetic polymer colloids (MPC) with spherical core–shell structures and poly(dimethyl diallyl ammonium chloride) (PDAC) were fabricated using the same approach [24]. Gradient thin films incorporating negatively charged magnetic colloidal particles and PDAC were fabricated through the layer-by-layer self-assembly technique. The assessment of their diagnostic potential involved the acquisition of T1- and T2-weighted images, with water as the testing medium. The signal intensity of T2-weighted images showed a decline with an increasing number of bilayers in the film gradients. These films have the potential to serve as a dipstick method for regular clinical diagnosis [25]. MPC and polyacrylic acid (PAA), featuring stimuli-sensitive cationic and anionic functional groups, respectively, were employed to generate thin-film gradients through the layer-by-layer technique. The film characteristics were manipulated by adjusting the pH and concentrations of the adsorbing solutions, resulting in the creation of gradient films consisting of 5.5, 10.5, and 15.5 bilayers. These newly developed thin film gradients were investigated for in vitro MRI, offering a quick lab-on-chip solution using a dipstick approach for highly sensitive in vitro biomolecular diagnosis [26].

This work emphasizes the exploitation of MRI for ultrasensitive in vitro molecular imaging of fluidic biological samples like blood, cell suspensions, and lysates. The fabrication of gradient thin films of core–shell magnetic-polymer colloids with variable compositions, sizes, surface charges, magnetic contents, and morphologies on the glass slides has been demonstrated. The gradient chip was explored as an imaging platform for surface-based MRI using pure water as a test liquid. This method potentially offers the facile sample application and disposable lab-on-chip as a dipstick approach for ultrasensitive in vitro molecular diagnosis of biological fluids via MRI.

## 2. Materials and Methods

### 2.1. Materials

PDAC, obtained from Aldrich in the form of 20% w/w solution, served as the polyelectrolyte for crafting thin films. The substrates employed were microscopic glass slides (25 × 75 × 1 mm, sourced from Globe Scientific Inc., USA). Glass substrates were meticulously cleaned using concentrated sulfuric acid (H_2_SO_4_) with a purity of 95%–98%, obtained from Sigma-Aldrich, and potassium dichromate (reagent grade, Scharlau). All utilized chemicals and materials were employed without any additional purification steps. Ultrapure water having a conductivity of 0.0055 µS and total dissolved solids (TDS) of 0.0, along with deionized water (DI), were utilized for solution preparation and washing procedures, respectively.

### 2.2. Synthesis of Magnetic Emulsion (ME) and Magnetic-Polymer Colloids

Magnetic-polymer colloids with isotropic core shells and directional asymmetry on two distinct halves (Janus morphologies) were prepared through seeded emulsion polymerization method. The oil-in-water ME, termed ME, was prepared following a process described elsewhere [27–30]. In brief, iron oxide nanoparticles (Fe_3_O_4_/Fe_2_O_3_) with oleic acid dispersion in octane were synthesized through the coprecipitation method. In this emulsion, the aqueous medium contained Triton X-405 and sodium dodecyl sulfate (SDS) as anionic surfactants to ensure the electrostatic stabilization of the ME.

To prepare magnetic-polymer colloids, an ME (50.0 g, with solid contents of 4.2%) was de-aerated by purging with N_2_ for 2 h. In the subsequent steps, 900 mg of monomers (styrene [St] and divinylbenzene [DVB]) and the initiator (4, 4′-azobis (4-cyanopentanoic acid) [ACPA]) were introduced into the ME. This mixture was heated to 70°C to initiate polymerization.

Various types of core–shell colloids were produced, including (i) functionalized colloids, which were obtained by adding methacrylic acid (MAA) monomer through an emulsion polymerization technique, resulting in the formation of a magnetic polymer designated as M-P(St/DVB/MAA). (ii) Negatively charged core–shell colloids derived from the as-prepared ME stabilized with SDS. (iii) Negatively charged ME coated with amino dextran (AMD) and (iv) Positively charged ME/AMD particles. Additionally, a Janus magnetic polymer colloid, M-PSt, was produced by adding a mixture of styrene monomers and the oil-soluble initiator 2,2′-azobis (2-isobutyronitrile) (AIBN) into the ME to initiate polymerization, as described above.

### 2.3. Characterizations of the Magnetic-Polymer Colloids

The zeta sizer (Malvern, Nano ZS) was employed to measure the hydrodynamic diameter (Dh) of the colloidal particles in their as-prepared state at 25°C. To ensure reproducibility, the experiments were conducted in triplicate. For a detailed examination of the colloidal particle morphology, a transmission electron microscope (TEM; Philips CM120) was utilized. In brief, a small amount of significantly diluted sample was placed on a carbon-coated copper grid, left to dry at room temperature, and then installed in the TEM for imaging.

### 2.4. Preparation of the Gradient LbL-SAMu Thin Films

**Figure 1:**
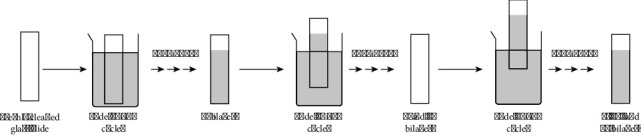
Schematic diagrams showing the preparation of the gradient layer-by-layer self-assembled multilayers (LbL-SAMu) thin films on the glass slides.

Before the application of the LbL-SAMu sequence, the glass microscope slides were immersed overnight in an acidic solution containing a 10% w/w potassium dichromate solution and concentrated H_2_SO_4_ in a 1:1 v/v ratio. The slides were then thoroughly rinsed in DI water and allowed to dry under ambient conditions. Freshly cleaned glass slides were dipped in a specific aqueous solution containing a known amount of cationic surfactant for 10 min, followed by a 10-min rinse in DI water. Subsequently, the slides with the first cationic layer were immersed in a distinct anionic particle dispersion for 10 min and then rinsed. Following the general scheme shown in Figure 1, the first bilayer was achieved in 40 min. To create another bilayer composite, the entire 40-min cycle was repeated without delay during the intermediate steps and drying. The slides were allowed to dry under ambient conditions after reaching the required number of bilayers. Aqueous dispersions of MPC were prepared in ultrapure water. Gradient thin films with four distinct regions were manually prepared using the LbL-SAMu technique.  Table 1 outlines the recipe for the preparation of the LbL-SAMu thin films. The gradient in thin films was generated by decreasing the immersion depth of slides in polyelectrolyte solutions after attaining the required number of bilayers. The regions of uncoated glass, 5, 10, and 15 bilayers, were prepared as illustrated in Figure 1.

### 2.5. Characterization of the Gradient LbL-SAMu Thin Films

The opacity of the gradient LbL-SAMu thin films was examined using an optical microscope (Optika B-600 MET) to compare the 5, 10, and 15-bilayer films. For detailed microstructural analysis, LbL-SAMu thin films were sputter-coated with gold using an atomic ion sputtering device, JEOL JFC-1500, to a thickness of 250 Å. Surface morphology, distribution of colloids, and surface occupancy of the thin films were examined in a low-vacuum analytical Scanning Electron Microscopy (SEM, JEOL JSM 6490LA) in a secondary electron imaging mode at an accelerating voltage of 20 kV.

The contact angle of the gradient LbL-SAMu thin films was measured using the sessile drop method. In brief, a 10–15 μL droplet of ultrapure water was produced at the tip of the syringe needle and dropped onto the thin film surface. This droplet was exposed to light, and a smart camera, positioned in alignment with the light source, captured the ensuing image. The contact angle, representing the angle formed between the interface of the liquid and vapor phases and the interface of the solid and liquid phases, was quantified through the utilization of the LB-ADSA plug-in within the ImageJ software.

The MRI analysis of the fabricated LbL-SAMu thin films was conducted using a clinical 1.5 T superconducting magnet, specifically the Toshiba Vantage Titan 1.5 T MRI machine in Japan. Integrated with a 71 cm aperture and featuring a 55 × 55 × 50 cm field of view (FOV), this system utilized Pianissimo technology to minimize acoustic noise. The assessment involved the utilization of T2 sequences, incorporating diverse values of MRI field echo (FE) sequences, specifically adjusting parameters TR (repetition time) and TE (echo time) to investigate signal intensity. T2-weighted images were acquired with TR set at 5000 ms, and TE values were varied at 15, 45, 75, 105, 135, and 165 ms to comprehensively assess T2 signal intensities. However, the T2-weighted images obtained at TE of 75 ms are presented in this manuscript. All the gradient LbL-SAMu thin films underwent testing under ambient conditions by immersing sets of three glass slides (per immersion) in ultrapure water within a specially crafted clear polyacrylic cell. The cell was subsequently positioned inside the multichannel body coil (as shown in the graphical abstract). The sagittal mode MRI images were analyzed using the iQ-VIEW PRO (Version 2.8.0.101) software integrated with the MRI machine and the K-PACS Workstation (Version 1.0.1) for statistical analysis. Uniform-sized regions of interest (ROI) were chosen across all sections of the gradient LbL-SAMu thin films, including 5, 10, and 15 bilayer sections, as well as a reference section from the noncoated area. The intensity data were computed based on the pixel count within each ROI. Mean intensity values were subsequently calculated for comparison, providing insight into the relative intensity counts of each thin film sample.

## 3. Results and Discussion

### 3.1. Characteristics of ME and Magnetic-Polymer Colloids

Magnetic-polymer colloids with core–shell and Janus morphologies were prepared via the seeded emulsion polymerization method using an oil-in-water ME [27–30].  Table 2 presents the details of the polymerization constituents, the size of prepared particles, and their respective morphologies.

Particles of various sizes were produced in the range of 170–320 nm. Due to their surface characteristics, the as-synthesized particles exhibited remarkable stability even during storage for more than 6 months. During the preparation of the ME, a nonionic surfactant (Triton X-405) and anionic SDS were employed for the emulsification of ME. The surface of the particles in the ME stabilized through the addition of Triton X-405 and SDS. Steric stabilization occurred as the octyl phenol functional groups of Triton X-405 were adsorbed onto the particle surface. Meanwhile, the hydrophilic ethoxylate molecular chains were oriented within the aqueous phase. The prepared microemulsion's surface displayed a negative charge, as indicated by the zeta potential value of −40 mV, suggesting the presence of sulfate functional groups from SDS on the ME particles.

**Figure 2:**
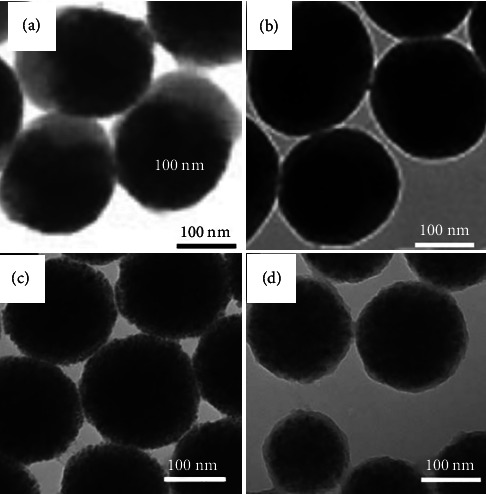
Core–shell morphology of the iron oxide nanoparticles (dark inner region) and a polymeric matrix (outer layer) of the (a) PDAC and M-PSt Janus particles, (b) PDAC and M-P(St/DVB/MAA) particles, (c) PDCA and ME particles, and (d) ME/AMD and ME particles.

For M-P (St/DVB/AA), the particle's surface was stabilized by the negatively charged carboxylate groups, which resulted from the addition of the anionic initiator, ACPA, and subsequent functionalization with the MAA monomer. The high stability of the as-prepared M-P(St/DVB/MAA) was verified through the negative value of zeta potential (∼−50 mV). The surface functionalization of the M-St Janus particles in the magnetic field improved their stability in an aqueous medium through surface modification of ME with AMD (designated as ME/AMD), resulting in the adsorption of positively charged amino groups on the particles, as confirmed by the positive zeta potential value (∼+35 mV). The confirmation of the core–shell structure of the synthesized/modified magnetic-polymer colloidal spherical particles was achieved through TEM analysis, as shown in Figure 2.

In the process of synthesizing the iron oxide/polymer nanocomposite particles, it was expected that the water-soluble initiator radicals would adhere to the surface of the monomer/magnetic droplet, promoting polymerization on the external surface of the iron oxide nanoparticles. Additionally, the presence of a crosslinking agent (DVB) within the monomer/magnetic droplet led to an uneven distribution of iron oxide nanoparticles within the polymer matrix, resulting in the formation of a core–shell morphology. It was observed that core–shell particles were produced when using a water-soluble initiator such as KPS and ACPA, whereas Janus particles were produced when using an oil-soluble initiator, namely AIBN. When an AIBN was present, polymerization initiated within the monomer/magnetic droplets. Resultantly, the iron oxide nanoparticles moved away from the droplet during polymerization because of the restricted miscibility of the polymer matrix with oleic acid on the surface of the magnetic nanoparticles (ME/AMD and ME particles), leading to the creation of anisotropic Janus morphology as shown in Figure 2(d).

### 3.2. Characteristics of Gradient LbL-SAMu Thin Films

**Figure 3:**
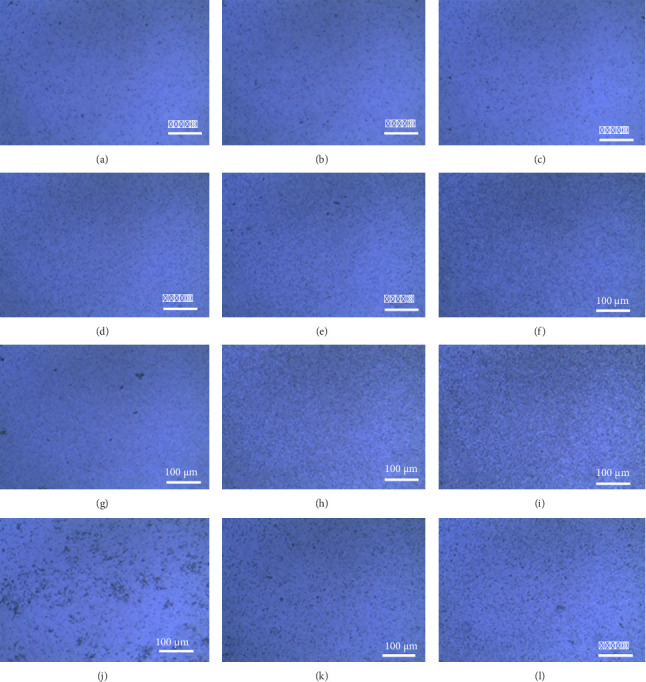
Optical microscopy of the gradient LbL-SAMu films showing the opacity of the glass slides (a–c) PDAC and M-PSt Janus particles, (d–f) PDAC and M-P(St/DVB/MAA) particles, (g–i) PDCA and ME particles, and (j–l) ME/AMD and ME particles (note: in this figure, from left to right, the films are composed of 5 bilayers, 10 bilayers, and 15 bilayers).

The gradient thin films, created through the LbL-SAMu approach, exhibited three distinct coating regions discernible even without instrumental aid. A decline in opacity from 15 bilayers to 5 bilayers was observed, as shown in the optical micrographs (Figure 3). The rise in opacity observed as the number of bilayers increased (from five to fifteen) within the gradient thin films was associated with a higher concentration of magnetic colloidal particles. Optical microscopy revealed an augmentation in surface area coverage corresponding to an increase in the number of bilayers across various sections of the gradient thin films. The colloidal particles were uniformly distributed in the gradient thin films containing positively charged (PDAC) and negatively charged colloids in the M-P(St/DVB/MAA) and ME films, as evident in the optical micrographs. However, the M-PSt Janus and oppositely charged colloids of alternating ME/AMD and ME gradient thin films exhibited the clustering of oppositely charged colloids within the gradient films, as highlighted by the darker regions in Figures 3(j), 3(k), 3(l).

**Figure 4:**
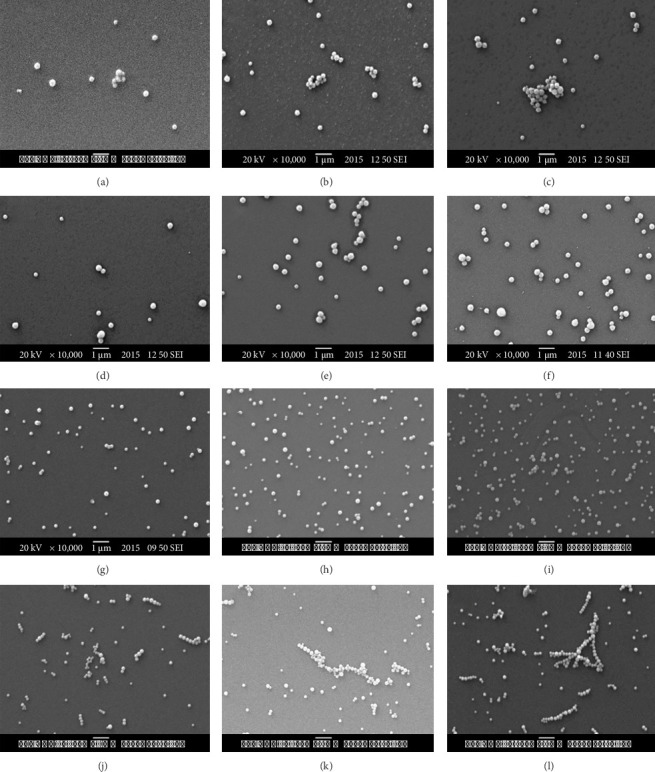
SEM micrographs of the gradient LbL-SAMu films (a–c) PDAC and M-PSt Janus particles, (d–f) PDAC and M-P(St/DVB/MAA) particles, (g–i) PDCA and ME particles, and (j–l) ME/AMD and ME particles. (Note: in this figure, from left to right, the films are composed of 5 bilayers, 10 bilayers, and 15 bilayers).

The distribution of colloidal particles in the gradient films was also confirmed by SEM, as shown in Figure 4. The gradient thin films of positively charged PDAC and negatively charged colloids, such as M-P(St/DVB/MAA) and ME (Figures 4(d), 4(e), 4(f), 4(g), 4(h), 4(i)), showed increasing adsorption of colloidal particles with a higher number of bilayers, which is in accordance with previous studies [25]. In the case of gradient thin films composed of PDAC and negatively charged magnetic colloids, successive bilayer additions were observed to result in increased particle adsorption in various sections of each film. This observation can be linked to the charge overcompensation effect, which guides the layer-by-layer growth of thin films, essentially increasing the concentration of components adsorbed in successive adsorption steps [31].

Some degree of agglomeration was observed in the gradient thin film of PDAC and Janus M-PSt colloids (Figures 4(a), 4(b), 4(c)). Aggregation of the particles that occurred through an increasing number of bilayers can be explained based on the non-uniform distribution of surface charges of particles, due to the asymmetric morphology of the adsorbing Janus particles. This growth pattern of the films can be attributed to the number of colloid particles that adsorb during the first deposition cycle and the subsequent deposition of oppositely charged colloids in the following dipping cycles. More uniform films can be achieved either by varying the substrate or by incorporating a polyelectrolyte after each deposition step or by increasing the intermediate washing steps between successive adsorption steps to produce a uniform charge distribution [32]. Therefore, uniformity in colloidal deposition steps ensures a uniform distribution of surface charges that increases the likelihood of deposition of a well-dispersed film. Relatively higher agglomeration was observed in the gradient thin films of oppositely charged colloids of ME/AMD and ME (Figures 4(i) and 4(j)) as compared to other gradient thin films. The growth pattern of the oppositely charged colloid films can be attributed to the number of colloid particles that adsorb during the first deposition cycle, as they serve as sites for electrostatic attraction and subsequent deposition of the oppositely charged colloid in the following dipping cycles.

**Figure 5:**
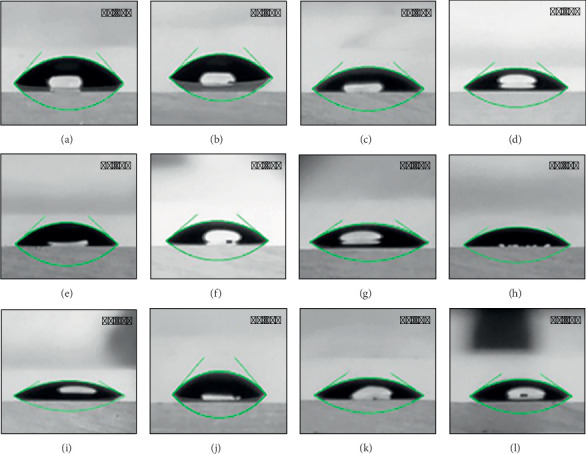
Contact angle values of the gradient LbL-SAMu films (a–c) PDAC and M-PSt Janus particles, (d–f) PDAC and M-P(St/DVB/MAA) particles, (g–i) PDAC and ME particles, and (j–l) ME/AMD and ME particles. (Note: in this figure, from left to right, the films are composed of 5 bilayers, 10 bilayers, and 15 bilayers).

A contact angle of less than 42° was consistently observed across the gradient thin films (Figure 5), indicating greater hydrophilicity that is essential for T2-weighted MRI contrast. A systematic, periodic reduction in the values of contact angle was noted with a rising number of bilayers in the gradient thin films of PDAC and negatively charged colloids, such as M-PSt Janus, M-P(St/DVB/MAA), and ME. This verifies the overcompensation of charge that drives the growth of LbL-SAMu thin films [33]. The growth of thin films of PDAC and negatively charged colloids is a consequence of charge reversal, which allows the surface to attract oppositely charged species in the ensuing steps of layer-by-layer assembly. The decrease in contact angle values can be correlated in a way that more charged particles are adsorbed with an increasing number of bilayers, resulting in greater attraction for water molecules in added bilayers due to more charged particles [34]. The decrease in contact angle, as shown by the film produced by oppositely charged colloids of ME/AMD and ME, is from 5 to 10 bilayer sections, that is, ∼40°–∼28°, but there is no significant difference in the subsequent bilayer, which is 40° and 28°, respectively. This is an important observation, as contact angle values indicate the extent of proton interaction of water molecules with the surface of produced films. Variations in such subtle interactions between water molecules can be highly significant in MRI applications [24]. It suggests the influence of aggregation of colloids into strings and clusters of particles as revealed in the SEM analysis (Figures 4(i) and 4(j)) of the surface morphology of the fabricated films.

**Figure 6:**
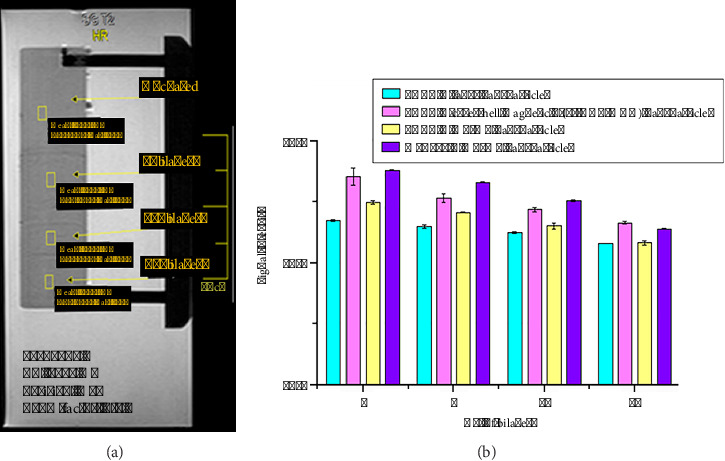
(a) A representative MRI scan of the gradient LbL-SAMu film depicting the mean signal intensity values of the ROI chosen in varied sections of a film. (b) Mean signal intensity graph of the actual GRADIENT LbL-SAMu films at TE = 75 ms and TR = 5000 ms. (Note: with an increase in the number of bilayers along the gradient, there is a declining trend in the intensities within the regions of interest [ROI]).

MRI is a molecular imaging technique acquired by targeting water molecules within a sample. The T2-weighted sagittal MRI of thin films showed four areas in gradient LbL-SAMu films: uncoated regions and 5, 10, and 15 bilayered sections. These gradient thin films were used for surface-based MRI using pure water as a test liquid. To further investigate the role of the contrast agent, three signal intensity values were recorded for each ROI, and their mean and standard deviation values were determined. The T2-weighted MRI of the gradient thin films showed a marked decline in intensities along the rising gradient, as shown in Figure 6(a). The mean signal intensity values of all ROIs were compared for each TE sequence of 15, 45, 75, 105, 135, and 165 ms.

A declining trend in mean signal intensity for each sample at all TE sequences with added bilayers was observed, as shown in Figure 6(b). Such a decline in signal intensity suggests amplifying negative contrast along the gradient because of rising particle concentration [35, 36]. The difference in intensity observed for various sections in the film was significant, and all areas were visible in MRI. This decline in signal intensity and hence enhanced negative contrast can be further improved by increasing the concentration of adsorbed particles in each section [37]. However, a noticeable drop in mean signal intensity values of gradient thin films of the ME/AMD and the ME particles implies greater contrast in classified sections. This greater difference in mean signal intensity values is attributed to both components as positively charged and negatively charged colloids in the film. The common observation in both films was the particle aggregation due to the interaction of magnetic particles and analytes that resulted in modification of the MRI signal, which provides the basis for molecular detection through MRI [38].

The M-PSt Janus colloid showed a relatively higher intensity drop or negative contrast through increasing bilayers of thin film by comparing with another magnetic colloid at the same gradient region values of TR and TE. This observation indicates that the Janus colloid was the best contrast agent among all tested samples of magnetic colloid in the present study (Figure 6(b)). Thin films of PDAC-M-PSt Janus nanoparticles exhibit overall low signal intensity at the same experimental conditions due to the asymmetric morphology. This suggests that one pole assists in adsorption due to the presence of charged functional groups and its ability to attract water molecules, while the other pole exhibits magnetic behavior. Based on this observation, it can also be deduced that the magnetic core size may affect the signal intensity. Since the size of the magnetic pole in Janus particles is significantly smaller than that of the polymeric pole, this indicates a size-dependent variation in magnetic behavior.

### 3.3. Statistical Analysis

**Figure 7:**
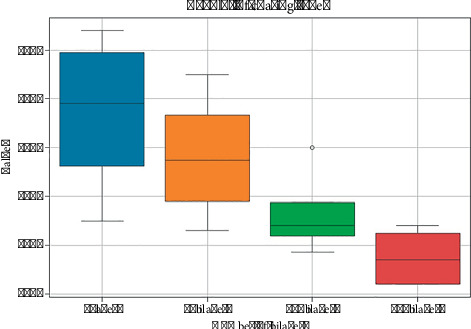
Statistical analyses of the correlation between the number of bilayer coatings and variation in signal intensity.

Python, a programming language, was employed to statistically analyze the correlation between the number of bilayer coatings and variations in signal intensity. Using Python-based statistical libraries, various parameters, including mean, variance, skewness, and kurtosis, were calculated for different sample conditions, as presented in Figure 7. Here, the mean represents that the average value for each condition has the central tendency in the values that tend to decrease from 0 layers (6337.5) to 15 bilayers (4875.0). Further, the variance shows broadness in the data points that spread around the mean. For example, 0 layers has the highest variance (785,625) to indicate more variability, while 15 bilayers have the lowest value (102,500) to reflect a more consistent dataset. Skewness measures the asymmetry of the data distribution, with positive values observed (e.g., 1.499 for 10 bilayers) to suggest a longer tail on the right, while negative values are noted (e.g., −0.483 for 0 layers) to indicate a tail on the left. Kurtosis reflects the “peakedness” of the distribution, with a higher positive value (2.657 for 10 bilayers) to suggest a sharp peak and heavy tails, whereas a strongly negative value (−5.518 for 15 bilayers) points to a distribution with flatter characteristics with light tails. Together, all these parameters assist in understanding the average behavior, the variability, and the shape of the data across different coatings fabricated.

**Figure 8:**
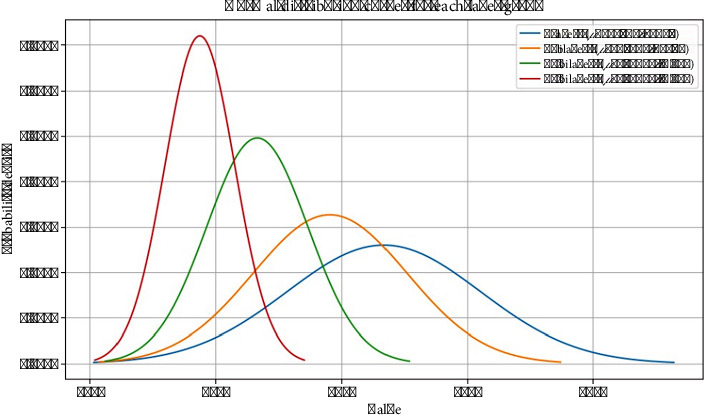
Normal distribution curves of intensity in correlation with numbers of layers.

The normal distribution curves of the signal intensity corresponding to the number of layers were analyzed using Python and represented in Figure 8. The curves of normal distribution show a decreasing trend in the values of the mean with the increase in the number of bilayers. The 0-layer group has the highest mean *μ* value of 6337.5, while the 15-bilayer group has the lowest mean *μ* value of 4875.0 to indicate a potential reduction in the measured intensity with an increase in the number of LbL-SAMus. Additionally, the standard deviation *σ* also decreases with more bilayers. For example, with 0 LbL layers, *σ* is noted as ≈ 768 to show the widest spread to imply higher variability. The 15 bilayers with *σ* ≈ 277 are the most consistent group with the narrowest distribution. This suggests that the increase in the number of bilayers not only lowers the central tendency but also improves consistency in the observed data.

## 4. Conclusions

In conclusion, this research highlights that colloidal Janus particles show improved negative contrast owing to their hydrophilic and magnetic pole in a 1.5 T clinical MRI scanner. The hydrophilic corona provides a protective shell for stabilization in aqueous media. Also, the resultant gradient thin film with significantly improved T2 contrast can be effectively utilized as a straightforward and reproducible method, particularly in the development of lab-on-chip devices. Moreover, these could serve as ultrasensitive molecular probes that can boost clinical MRI for in vitro diagnosis of biological samples like fluids or cellular imaging. This can further be refined for the cellular visualization process, and even the use of these films in microfluidic lab-on-chip MRI systems has been proposed. These films with specificity toward certain cells can also be fabricated by engineering magnetic colloids with specific cell receptors. Conclusively, the adsorbed films can be used for in vitro diagnostic applications as a dip-stick approach with a slight improvement/modification in both the phantoms and the instrument. Although this study is experimental, advanced numerical methods such as compact finite difference and alternating direction implicit (ADI) schemes could be beneficial for modeling thin-film behavior and signal transport in lab-on-chip systems. These techniques may be applied in future work to optimize the film's response under external stimuli.

## Figures and Tables

**Table 1:** The recipe for the magnetic-polymer colloids LbL-SAMu thin films.

Sample	Composition of LbL-SAMu thin film		Cationic dispersion (% w/w)^a^	Anionic dispersion (% w/w)^a^
	Cationic species	Anionic species		
1	PDAC	M-PSt Janus	0.035	0.35
2	PDCA	M-P(St/DVB/MAA)	0.035	0.35
3	PDCA	ME	0.03	0.30
4	ME/AMD	ME	0.30^a^	0.30^a^

^a^Dispersion was prepared in DI water.

**Table 2:** Details of the components added during polymerization, particle size, magnetic content, and morphology of the prepared particles.

Samples	Initiator	Surface modification	Surface charge	Particle size (nm)	Morphology^∗^
M-PSt	AIBN	SDS/PPA	Negative	242	Janus
M-P(St/DVB/MAA)	ACPA	MAA	Negative	320	Core–shell
ME	—	SDS	Negative	191	Core–shell
ME/AMD	—	AMD	Positive	176	Core–shell

^∗^Morphology was determined through TEM analysis.

## Data Availability

The data supporting the findings of this study are available from the authors upon reasonable request.
